# Sleep Quality and Its Sociodemographic, Behavioural, Clinical, and Regional Correlates Among Adults in Kazakhstan: A National Cross-Sectional Survey

**DOI:** 10.3390/clockssleep8020034

**Published:** 2026-06-12

**Authors:** Yerlan Ismoldayev, Anel Ibrayeva, Alfiya Shamsutdinova, Marat Shoranov, Bolat Sadykov, Altynay Sadykova, Timur Saliev, Shynar Tanabayeva, Ildar Fakhradiyev

**Affiliations:** 1 Department of Medicine, S.D. Asfendiyarov Kazakh National Medical University, Almaty 050000, Kazakhstan; 2 College of Medicine, Korea University, Seoul 02841, Republic of Korea

**Keywords:** sleep–wake disorders, sleep quality, cross-sectional studies, adult, risk factors, Kazakhstan

## Abstract

Population-based evidence on sleep quality in Kazakhstan remains limited. This study describes sleep quality as a multidimensional construct among adults in Kazakhstan using data collected during the first national survey wave after the adoption of a single national time zone. The survey was designed as a national post-transition baseline assessment and not as an evaluation of the causal impact of the time-zone reform. Associations with socio-demographic, behavioural, clinical, and regional factors were examined. We conducted a nationally representative cross-sectional survey of adults aged 18–69 years in Kazakhstan from May to October 2025 using a multistage stratified cluster design. Sleep quality was assessed with the Pittsburgh Sleep Quality Index (PSQI). Poor sleep quality was defined as a global PSQI score > 5. Complete PSQI data were available for 5872 participants. Descriptive analyses examined the global PSQI score and the seven component scores. Survey-weighted multivariable logistic regression was used to identify factors independently associated with poor sleep quality. The weighted prevalence of poor sleep quality was 28.1%, and the weighted mean global PSQI score was 4.43. The greatest component burden was attributable to sleep latency (mean 0.87), subjective sleep quality (0.82), and sleep disturbances (0.80), whereas use of sleep medication contributed minimally (0.11). Poor sleep quality was more common among women, older adults, urban residents, and participants with diabetes, current smoking, heavy episodic drinking, and depressive symptoms. In the adjusted model, female sex (aOR 1.37, 95% CI 1.19–1.57), age 55 years or older versus 18–24 years (1.98, 1.53–2.55), diabetes (1.47, 1.22–1.78), current smoking (1.28, 1.10–1.50), heavy episodic drinking (1.43, 1.16–1.76), and depressive symptoms (4.26, 3.52–5.15) were independently associated with higher odds of poor sleep quality. Rural residence was inversely associated with the outcome (0.71, 0.61–0.84). Compared with the North, higher odds were observed in the Central region (2.00, 1.46–2.74), East (1.94, 1.48–2.53), West (1.48, 1.17–1.88), and Almaty city (2.18, 1.72–2.76). Poor sleep quality is common among adults in Kazakhstan and is characterized primarily by difficulties with sleep initiation, perceived sleep quality, and nocturnal disturbances. The findings provide national post-transition baseline evidence and suggest that sleep health surveillance in Kazakhstan should prioritize demographic, mental health, behavioural, and regional inequalities while avoiding causal interpretation of the time-zone reform itself.

## 1. Introduction

Sleep is a fundamental physiological function and one of the key components of public health. Chronic sleep deprivation and poor sleep quality are associated with impaired daytime functioning, reduced cognitive performance, an increased risk of psychoemotional disorders, as well as adverse cardiometabolic consequences [1]. In recent years, sleep has increasingly been regarded not as a merely individual behavioral characteristic, but as an independent population health factor with both clinical and preventive significance [2].

Population-based studies conducted in different countries show that sleep disturbances are widespread among adults; however, their prevalence varies substantially according to age, sex, region of residence, educational level, psychoemotional status, and the presence of chronic diseases. For example, in a large nationwide study conducted in China, the prevalence of sleep disturbances was 19.2% in a sample of 94,454 adults, with pronounced variation across provinces [3]. In another population-based study among adults living in Southern Brazil, poor sleep quality was identified in 32.7% of participants; a higher risk was observed among women, smokers, respondents with depression, polypharmacy, and poor self-rated health [4]. Among middle-aged and older Chinese adults, poor sleep quality was also shown to be common and associated with older age, female sex, lower educational attainment, chronic diseases, and unfavorable behavioral factors [5].

Alongside individual and clinical factors, sleep quality is also shaped by the broader temporal context in which a person lives. Contemporary circadian literature emphasizes the importance of alignment between biological time, solar time, and social time. Their misalignment may be accompanied by sleep disturbances, daytime sleepiness, reduced work capacity, and other adverse consequences [6]. In this context, particular attention has been given to the phenomena of circadian misalignment and social jetlag, as well as to the role of official time organization in population health. The position of the American Academy of Sleep Medicine emphasizes that standard time is most closely aligned with human circadian biology and is therefore preferable from the standpoint of health and safety [7].

For Kazakhstan, this issue became especially relevant after the introduction of a unified time zone, UTC+5, across the country on 1 March 2024, when in most regions the clocks were moved one hour back [8]. Officially, this decision was justified by the need to unify time across the national territory and, according to statements by government authorities, by the greater alignment of the new regime with natural solar cycles [9]. At the same time, the reform triggered broad public discussion, including concerns related to well-being, wakefulness, and sleep among the population.

Despite the public importance of this issue, data on sleep disturbances in Kazakhstan remain limited. According to the available publications, sleep research in the country has thus far focused mainly on specific clinical and occupational groups. For example, among patients with type 2 diabetes in Astana, insomnia was found to be associated with anxiety and depression, while among emergency medical service workers in the East Kazakhstan and Abai regions, a high prevalence of insomnia, anxiety, and depression was reported. However, large population-based studies of sleep quality among the adult population of Kazakhstan, comparable to international population-based surveys, are scarcely represented in the available literature [10,11].

This gap is particularly important because the available evidence does not yet allow sleep quality in Kazakhstan to be evaluated as a population-level public health indicator. Previous clinical and occupational studies have provided valuable data for selected groups, but they do not capture the distribution of sleep problems across the general adult population, nor do they describe how sleep quality varies by socio-demographic, behavioural, clinical, and regional characteristics. A nationally representative assessment is therefore needed to determine the burden and structure of poor sleep quality in Kazakhstan and to place these findings within the broader context of sleep epidemiology in Central Asia. Such evidence is also relevant for future public health monitoring. Importantly, the timing of the survey should be interpreted only as defining the post-transition context in which the baseline assessment was conducted. Because no comparable pre-reform sleep quality data were collected within the same study framework, the present analysis cannot determine whether the 2024 time-zone reform changed sleep quality. Rather, it provides nationally representative reference data on sleep quality after the transition and identifies population groups and regions with higher sleep-health burden.

Thus, the present study aimed to assess sleep quality among the adult population of Kazakhstan and to analyze its associations with socio-demographic, behavioural, clinical, and regional factors.

## 2. Results

### 2.1. Demographic Characteristics

The analytical sample included 6712 participants aged 18–69 years (mean age 41.6 ± 14.0 years; median 40 years) (Table 1). Women constituted 52.4% (95% CI 51.2–53.6) of the study population, and 63.0% (95% CI 61.8–64.2) resided in urban areas. Participants of Turkic ethnicity represented 76.0% (95% CI 74.9–77.0), and 58.6% (95% CI 57.4–59.8) had completed higher education. With respect to health-related characteristics, 66.5% of participants had overweight or obesity, 27.7% (95% CI 26.7–28.9) were hypertensive, 12.2% (95% CI 11.4–13.0) had diabetes, 24.4% (95% CI 23.4–25.5) reported current smoking, and 10.2% (95% CI 9.5–10.9) reported heavy episodic drinking. Symptoms consistent with depressive symptomatology were observed in 11.4% of participants, whereas 33.5% were classified as having insufficient physical activity.

### 2.2. PSQI Structure and Domain-Specific Burden

Complete PSQI data were available for 5872 participants (87.5%) (Table 2). Among participants with complete PSQI data, the weighted mean global PSQI score was 4.43 (95% CI 4.36–4.50). The unweighted median global PSQI score was 4, with an interquartile range of 3–6. Overall, 1661 participants were classified as having poor sleep quality (PSQI > 5), corresponding to a weighted prevalence of 28.1%; the remaining 4211 participants were classified as having good sleep quality, corresponding to 71.9%.

Cronbach’s alpha for the seven PSQI components was 0.51, indicating modest internal consistency. This finding was interpreted in light of the multidimensional structure of the PSQI, in which the seven components reflect related but clinically distinct aspects of sleep rather than interchangeable items of a single homogeneous scale [12,13]. Previous psychometric reviews and factor-analytic studies have shown that the PSQI may have variable multidimensional structures across populations, and that individual components can contribute differently to the global score [14,15,16]. Therefore, in the present study, the global PSQI score was used as the primary established measure of subjective sleep quality, whereas component scores were interpreted descriptively to characterize the domain-specific burden of sleep problems.

Examination of the seven PSQI components showed that the greatest mean burden was attributable to sleep latency (mean 0.87), subjective sleep quality (0.82), and sleep disturbances (0.80). In contrast, use of sleep medication contributed minimally (mean 0.11) (Table 3). Any degree of impairment (component score ≥ 1) was most frequent for sleep disturbances (73.9%), subjective sleep quality (69.1%), and sleep latency (63.2%), whereas moderate-to-severe problems (score ≥ 2) were most common for sleep latency (18.8%) and habitual sleep efficiency (18.2%). When the summed component burden was decomposed, sleep latency, subjective sleep quality, and sleep disturbances together accounted for more than half of the total PSQI component burden.

### 2.3. Bivariate Distribution of Poor Sleep Quality

**Figure 1:**
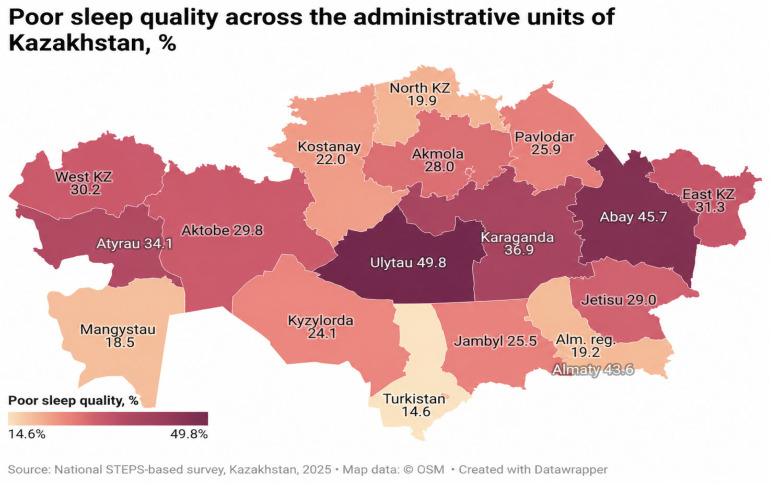
Weighted prevalence of poor sleep quality (PSQI > 5) across the 17 regions and 3 cities of republican significance of Kazakhstan.

Poor sleep quality was more prevalent in women than in men (32.1% vs. 24.7%) and increased progressively with age, from 21.5% in participants aged 18–24 years to 35.0% in those aged 55 years or older (Table 4). Urban residents had a higher prevalence than rural residents (32.2% vs. 20.8%). Marked regional heterogeneity was observed, with the highest prevalence in Almaty city (43.6%), the Central macro-region (39.1%), and the East (37.7%), and the lowest prevalence in Shymkent city (18.6%) and the South (20.9%). A similar pattern was seen for the weighted mean global PSQI score, which ranged from 3.69 in Shymkent city and 3.99 in the South to 5.14 in the Central region and 5.55 in Almaty city. Poor sleep quality was also more common among participants with obesity (31.3%), hypertension (34.4%), diabetes (40.8%), current smoking (31.0%), heavy episodic drinking (37.3%), and especially depressive symptoms (59.7%) (Table 5). By contrast, differences by education were minimal. Poor sleep quality was also slightly more common among participants with sufficient than insufficient physical activity (29.1% vs. 25.4%). Because the physical activity indicator combined work-related, transport-related, and leisure-time activity, this crude difference should be interpreted cautiously and does not necessarily reflect an adverse association of leisure-time physical activity with sleep quality. The weighted prevalence of poor sleep quality also showed marked variation across individual administrative units of Kazakhstan (Figure 1).

### 2.4. Multivariable Correlates of Poor Sleep Quality

Among the 5872 participants with complete PSQI data, 5465 were included in the multivariable regression model and 407 were excluded because of missing data on one or more covariates. Included and excluded participants were broadly similar in diabetes status and heavy episodic drinking, whereas excluded participants had a somewhat higher prevalence of poor sleep quality (34.6% vs. 27.8%) and were more likely to have missing information on depressive symptoms, BMI, place of residence, education, smoking, and sex (Supplementary Table S1).

In the weighted multivariable logistic regression model, female sex remained independently associated with higher odds of poor sleep quality (aOR 1.37, 95% CI 1.19–1.57) (Table 6). Compared with participants aged 18–24 years, the odds were higher in those aged 35–44 years (aOR 1.30, 95% CI 1.02–1.66), 45–54 years (1.47, 1.14–1.90), and 55 years or older (1.98, 1.53–2.55). Diabetes (1.47, 1.22–1.78), current smoking (1.28, 1.10–1.50), heavy episodic drinking (1.43, 1.16–1.76), and depressive symptoms (4.26, 3.52–5.15) were also independently associated with poor sleep quality. Rural residence was inversely associated with the outcome (aOR 0.71, 95% CI 0.61–0.84). Region remained an important independent correlate: compared with the North, higher odds of poor sleep quality were observed in the Central region (2.00, 1.46–2.74), East (1.94, 1.48–2.53), West (1.48, 1.17–1.88), and Almaty city (2.18, 1.72–2.76). Education, BMI, blood pressure status, and physical activity were not independently associated with poor sleep quality after adjustment.

As a sensitivity analysis, we refitted the multivariable logistic regression model among all participants with complete PSQI data, retaining missing covariate values as separate “missing” categories. The results were broadly consistent with the complete-case model. The direction and magnitude of the key associations remained similar, including those for female sex, older age, diabetes, current smoking, heavy episodic drinking, depressive symptoms, rural residence, and regional differences. This suggests that the complete-case restriction did not materially alter the main conclusions. Detailed results of the sensitivity analysis are presented in Supplementary Table S2.

## 3. Discussion

This study adds national population-based evidence on sleep quality in Kazakhstan, a setting for which epidemiological sleep data have been scarce. The overall burden observed in our survey appears to fall within the broad range reported in community-based studies from middle-income and upper-middle-income settings rather than representing an obvious outlier. This is important because international evidence suggests that estimates of poor sleep quality vary substantially across countries and study designs, and that low- and middle-income countries remain underrepresented in sleep surveillance. In that sense, our findings contribute not only national data, but also a regional gap in the broader global sleep-health literature [3,17].

An important contribution of the present study is that sleep was examined as a multidimensional construct rather than only through a dichotomous “poor sleeper” definition. The component profile suggests that the main burden is concentrated in difficulties related to sleep initiation, perceived sleep quality, and nocturnal disruption, whereas sleep medication contributes relatively little. This pattern is consistent with the broader PSQI literature, in which sleep latency and sleep disturbance repeatedly emerge as especially informative domains, both in general-population psychometric work and in studies linking individual PSQI components with metabolic and functional outcomes. Taken together, this supports the view that, at population level, sleep problems are often driven less by pharmacological dependence and more by difficulties falling asleep and maintaining consolidated sleep [1,16,18,19].

The sex and age gradients observed in our data are also consistent with previous population-based studies. Higher vulnerability among women has been described in community samples from Brazil, Japan, China, and Germany, while increasing sleep problems with age are among the most reproducible findings in sleep epidemiology. These regularities likely reflect the combined influence of biological, psychosocial, and clinical pathways, including menopausal transition, greater burden of chronic disease, caregiving roles, and cumulative psychosocial stress. At the same time, the absence of an independent educational gradient after adjustment suggests that in Kazakhstan, sleep inequality may be shaped more strongly by contextual, behavioural, and health-related factors than by formal schooling alone. This point is noteworthy because the literature on education and sleep is mixed: some studies report a gradient, whereas others show attenuation after fuller adjustment [4,16,20,21].

The strongest association in our model was with depressive symptoms, which is in line with a large body of evidence showing a close and probably bidirectional link between disturbed sleep and affective distress. Longitudinal meta-analytic evidence indicates that insomnia substantially increases the later risk of depression, while cross-sectional studies using PSQI likewise show that poorer subjective sleep is tightly coupled with depressive symptomatology. In practical terms, this means that poor sleep in the general population should not be treated as an isolated complaint: it is also a marker of psychological vulnerability. For Kazakhstan, this has immediate implications for screening, because sleep complaints may help identify individuals with otherwise under-recognized emotional distress in primary care and community settings [22,23].

The independent associations with diabetes, current smoking, and heavy episodic drinking also align with previous research. In people with type 2 diabetes, poor sleep quality has been repeatedly linked to poorer glycaemic status and to a greater overall psychosocial burden; notably, Kazakhstan-based clinical data already show that sleep problems in adults with diabetes cluster with anxiety and depression [10,24]. Likewise, smoking has been associated with worse sleep quality in both population-based studies and clinical research, while alcohol appears to impair subjective sleep even when its short-term sedative effects might suggest otherwise [25,26]. The fact that obesity and blood pressure status were not independently associated after mutual adjustment is also defensible in light of prior work showing that some cardiometabolic correlates weaken once mental health, comorbidity, and other behavioural factors are taken into account [27,28].

The seemingly inverse pattern for physical activity should be interpreted cautiously. In the bivariate analysis, poor sleep quality was slightly more common among participants classified as sufficiently active, but this association disappeared after multivariable adjustment. This finding should not be interpreted as evidence that leisure-time exercise worsens sleep. In this study, physical activity was operationalized as total MET-min/week across work-related, transport-related, and leisure-time domains. Therefore, participants classified as sufficiently active may have included individuals whose activity was largely occupational or transport-related rather than recreational. Domain-specific activity patterns may have different relationships with sleep, depending on timing, intensity, physical strain, and recovery. Previous evidence suggests that occupational physical activity and late or strenuous activity may be associated with poorer sleep outcomes, whereas total activity volume alone may mask these differences [29,30,31]. Thus, the crude bivariate pattern in our data may partly reflect domain heterogeneity, while total physical activity was not independently associated with poor sleep quality after adjustment.

One of the most analytically interesting findings is the persistence of urban and regional heterogeneity after adjustment for individual-level characteristics. Comparable studies from China have also documented substantial between-province variation, and research comparing rural and urban populations suggests that differences in light exposure, daily schedules, and circadian organization may contribute to distinct sleep patterns [3]. In addition, meta-analytic evidence indicates that exposure to light at night is associated with worse sleep outcomes, offering one plausible pathway through which urban environments may affect sleep [32]. These findings argue against reducing sleep quality to an individual lifestyle issue alone; instead, they point to the role of living environment, social timing, and ecological conditions [33].

The higher prevalence of poor sleep quality in Almaty city and in the Central and Eastern regions may reflect the combined influence of several contextual factors rather than a single determinant. Importantly, these regional associations may be affected by unmeasured confounding. For example, shift work and irregular work schedules may be unevenly distributed across regions and may influence circadian alignment and sleep timing [6]. Chronotype and social timing may also differ between populations and could modify how individuals adapt to local schedules and light–dark conditions [34]. Environmental light exposure, including artificial light at night in large urban settings, may be another relevant pathway, particularly in densely urbanized areas [32]. Occupational stress and detailed socioeconomic conditions may also influence sleep through psychosocial strain, housing conditions, commuting patterns, and access to health-related resources. Although the analysis adjusted for several individual-level socio-demographic, behavioural, and clinical factors, the absence of direct measures of shift work, chronotype, environmental light exposure, occupational stress, and detailed socioeconomic status means that the regional findings should be interpreted as hypothesis-generating markers of spatial variation in sleep health rather than as evidence of specific regional causes.

Because the survey was conducted after Kazakhstan adopted a single national time zone (UTC+5), the spatial pattern should be interpreted within a post-transition temporal context. However, this context should not be conflated with evidence of a time-zone effect. Previous sleep-health literature supports the relevance of standard time for circadian alignment and health [7], while light exposure [32] and social jetlag [34] are also recognized as important factors related to sleep outcomes. Nevertheless, the cross-sectional design, the absence of comparable pre-reform sleep measurements, and the lack of repeated follow-up data preclude causal attribution of the observed regional differences to the 2024 reform. Therefore, the present study should be understood as a national post-transition baseline for future sleep-health surveillance rather than as an impact evaluation of the time-zone change. A direct assessment of the reform would require longitudinal or quasi-experimental pre-post studies with comparable sleep measures, including chronotype, social jetlag, and light exposure.

From a public health perspective, the findings support moving sleep closer to the core of noncommunicable disease surveillance in Kazakhstan. Recent global reviews argue that sleep health should be treated as a pillar of health policy alongside nutrition and physical activity, yet many countries still lack standardized population data [2]. Our results suggest that future surveillance and prevention efforts should prioritize groups in whom sleep problems are likely to cluster with broader vulnerability: women, older adults, urban residents, people with depressive symptoms, people with diabetes, and those exposed to tobacco and harmful alcohol use. At the same time, the multidimensional PSQI profile indicates that interventions focused on sleep timing, sleep initiation, and fragmentation may be more relevant than a narrow focus on medication use alone.

Finally, several limitations should be considered when interpreting these findings. First, the study was cross-sectional, so the observed associations cannot be interpreted causally. Second, sleep quality was assessed using a validated self-report instrument rather than objective measures such as actigraphy or polysomnography; therefore, self-report and recall bias cannot be excluded. Cultural differences in the perception and reporting of sleep quality may also have influenced PSQI responses. Third, the internal consistency of the seven PSQI components was modest, which likely reflects the multidimensional structure of the PSQI, as it captures related but distinct aspects of sleep rather than a single homogeneous construct. Fourth, the survey did not collect data on occupational shift work, chronotype or social jetlag, environmental light exposure, occupational stress, or detailed socioeconomic status. These unmeasured factors may have confounded or mediated the observed urban and regional associations with sleep quality. Therefore, residual confounding cannot be excluded, and regional differences should be interpreted cautiously. Seasonal variation may also have affected sleep patterns, as data collection was conducted between May and October. Fifth, although complete PSQI data were available for 5872 participants, the fully adjusted regression model included 5465 complete cases; thus, 407 participants were excluded because of missing covariate information. Missingness cannot be assumed to be completely random, as excluded participants had a somewhat higher prevalence of poor sleep quality and differed from included participants in the pattern of missing covariate data. Complete-case analysis was chosen as a transparent primary approach because the proportion of excluded participants was relatively small and because multiple imputation would have required assumptions about the missing-data mechanism that could not be reliably verified in this cross-sectional survey with a complex sampling design. In addition, the study was not designed as a pre-post evaluation of the time-zone change. Despite these limitations, it provides rare nationally representative evidence on sleep quality in Central Asia and offers an empirical basis for future monitoring of sleep health in Kazakhstan.

## 4. Materials and Methods

### 4.1. Study Design and Sample

We conducted a cross-sectional, nationally representative survey of adults aged 18–69 years in Kazakhstan from May to October 2025 using a multistage stratified cluster design. This manuscript was prepared in accordance with the Strengthening the Reporting of Observational Studies in Epidemiology (STROBE) reporting guideline for cross-sectional observational studies. Field implementation covered all 17 regions of Kazakhstan and the three cities of republican significance (Almaty, Astana, and Shymkent). For the purposes of analysis, the territorial structure was prespecified in terms of eight analytical geographic domains: five macro-regions (North, Central, East, South, and West) and three cities analysed separately (Astana, Almaty, and Shymkent). A total of 6720 individuals were selected. Eight records with reported age > 69 years, which fell outside the WHO STEPS target age range, were excluded, yielding a final analytic sample of 6712 participants. Survey weights were applied, and the complex sampling design, including stratification and clustering, was accounted for in all national estimates and regression analyses.

### 4.2. Sampling

A multistage cluster sampling design with stratification by sex (male, female) and age group (18–24, 25–34, 35–44, 45–54, and ≥55 years) was used. The sample size was calculated using the World Health Organization STEPS sample size calculator (Excel-based tool) for each of eight prespecified analytical geographic domains. Assuming a 95% confidence level (Z = 1.96), an expected prevalence of 50%, a margin of error of 5%, a design effect of 1.5, and an anticipated response rate of 70%, the required sample size was 823 participants per domain. Across eight domains, this corresponded to a minimum total sample size of approximately 6585 participants. To facilitate balanced field allocation, maintain adequate representation across domains, and compensate for non-response, the final target sample size was set at 6720 participants.

**Figure 2:**
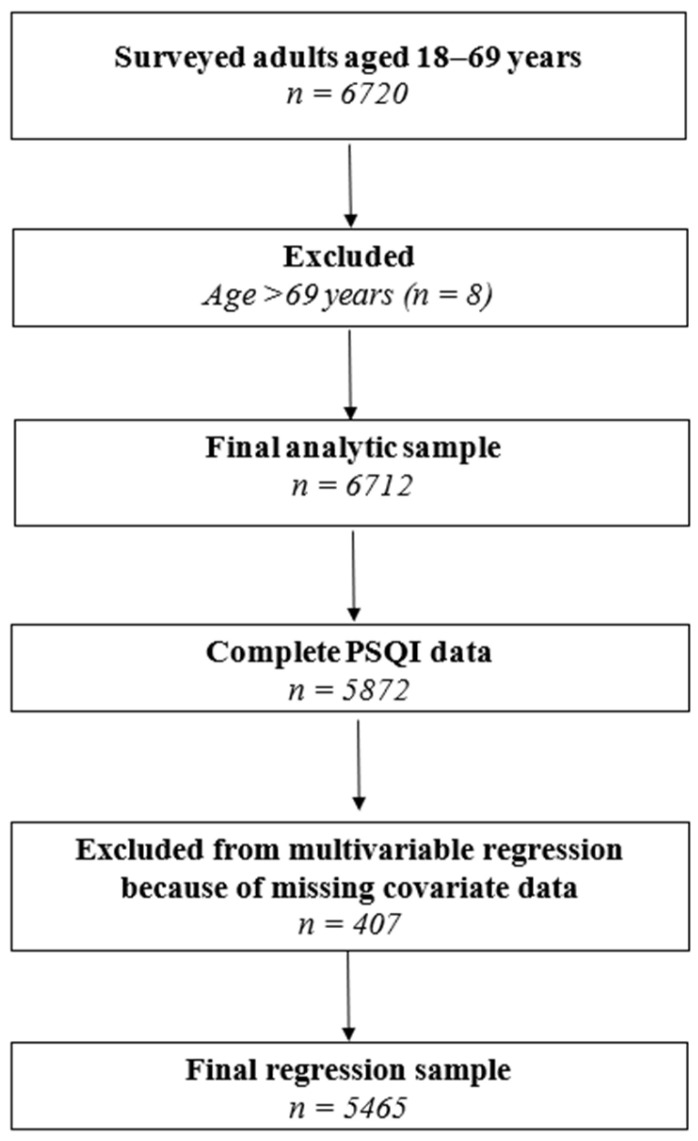
Flowchart of participant inclusion in the analytic sample of 6720 surveyed adults, 6712 were eligible for analysis, 5872 had complete PSQI data, and 5465 were included in the final multivariable regression model.

Sampling was implemented in three stages. At the first stage, primary sampling units (PSUs), defined as districts and major urban centres, were selected with probability proportional to size using official population data from the Bureau of National Statistics of the Republic of Kazakhstan. At the second stage, primary health care (PHC) facilities within each selected PSU were chosen as secondary sampling units in proportion to the size of the registered population, based on the national PHC registry. At the third stage, households within each selected PHC catchment area were randomly selected using the Randhold.xls tool, and one eligible adult per household was chosen using the Kish method. The planned number of households per PHC was approximately 28 (Figure 2).

### 4.3. Data Collection

Before the survey, data collection teams received training on interview techniques and physical/biochemical measurements. Interviewers explained the study’s goals to each household and obtained informed consent. Face-to-face interviews were conducted, and physical/biochemical measurements were taken on the same day.

### 4.4. Data Variables

The study followed the standardized WHO STEPwise approach [35]. The questionnaire was based on the standard WHO STEPS instrument and adapted for the Kazakhstan survey context. It was administered in Russian or Kazakh according to participants’ preference. In both cases, items were adapted from the original English WHO STEPS instrument using a standard forward–backward translation procedure. First, two bilingual physicians independently translated the English questionnaire into Russian and Kazakh, respectively, and any discrepancies between the forward translations were reconciled into single Russian and Kazakh versions. These preliminary versions were then back-translated into English by independent bilingual translators. The back-translations were compared with the original English questionnaire, and iterative revisions were undertaken until the research team agreed that both the Russian and Kazakh versions were conceptually equivalent to the source instrument.

In Step 1, trained interviewers collected socio-demographic data (age, sex, ethnicity, place of residence, education level, marital status, occupation) and information on behavioral risk factors (tobacco use, alcohol consumption). Steps 2–3 included physical measurements and venous blood sampling.

Depressive symptoms were derived from the WHO STEPS Mental Health/Depression module. A positive screening variable was defined by affirmative responses to the two core symptoms: depressed mood and/or loss of interest or pleasure during the reference period. Module symptoms were summed to create a symptom count, excluding the sleep-related item to avoid overlap with the PSQI outcome. Participants with a positive screening variable and five or more symptoms in the modified count were classified as having depressive symptoms. This indicator was interpreted as a population-level symptom measure rather than a clinical diagnosis of depression.

Physical activity was derived from the WHO STEPS physical activity module. Total physical activity was calculated by combining work-related, transport-related, and leisure-time activity and expressed as MET-min/week. Participants were classified as having insufficient physical activity if their total physical activity was <600 MET-min/week, in accordance with WHO recommendations. Those with ≥600 MET-min/week were classified as sufficiently active. This variable therefore reflected overall activity volume across domains and should not be interpreted as leisure-time exercise alone.

Region of residence was originally recorded according to all administrative units of Kazakhstan, including regions and cities of republican significance. In line with the prespecified sampling and analytical framework, territorial variation was examined across eight analytical geographic domains: five macro-regions (North, Central, East, South, and West) and three cities analysed separately (Astana, Almaty, and Shymkent). The three cities were retained as distinct categories because of their specific urban and administrative profiles, whereas the remaining administrative units were grouped into macro-regions to support interpretable presentation and stable design-based estimates in regression analyses. In addition, for descriptive geographic visualization, the weighted prevalence of poor sleep quality was presented across all administrative units of Kazakhstan.

Anthropometry followed WHO STEPS SOPs. Height was measured with a portable stadiometer to 0.1 cm, weight with a calibrated digital scale to 0.1 kg (light clothing, no shoes), and waist/hip circumferences with a non-stretch tape to 0.1 cm. Each measure was taken twice (third if discrepancy >0.5 kg or >0.5 cm) and averaged.

Blood pressure was measured using an automated oscillometric device A&D UA-888. After ≥5 min of seated rest (no caffeine/smoking/exercise ≥30 min), three readings were obtained at 1 min intervals; the mean of the 2nd and 3rd readings (mmHg) was used for analysis.

The biochemical panel included HbA1c, triglycerides, total cholesterol, HDL-cholesterol, and LDL-cholesterol. Venous blood was collected by trained staff according to laboratory SOPs; HbA1c was analyzed from EDTA whole blood. Lipids were assayed by standard enzymatic colorimetry; and LDL-C was calculated using the Friedewald equation, and computed only when TG < 4.5 mmol/L. LDL-cholesterol was used for descriptive characterization only and was not included as a key predictor in regression analyses.

Body mass index (BMI) was calculated and categorized into underweight (<18·5 kg/m^2^), normal weight (18·5–24·9 kg/m^2^), overweight (25–29.9 kg/m^2^), and obesity (≥30 kg/m^2^). Blood pressure status was categorized as normotension (SBP < 120 mmHg and DBP < 80 mmHg), pre-hypertension (SBP 120–139 mmHg and/or DBP 80–89 mmHg), and hypertension (SBP ≥ 140 mmHg and/or DBP ≥ 90 mmHg and/or use of antihypertensive medication within the previous 2 weeks). The diabetes status was classified as follows: no diabetes: glycated hemoglobin (HbA1c) < 6.5% and no use of diabetes medication within the past 2 weeks and no current insulin use for diabetes; diabetes: HbA1c ≥ 6.5% and/or use of diabetes medication within the past 2 weeks and/or current insulin use for diabetes.

Current smoking was defined as a “yes” response to current tobacco use; HED was defined as ≥60 g of pure alcohol on at least one occasion in the past 30 days (WHO definition).

### 4.5. Sleep Quality (PSQI)

To assess sleep quality, the Pittsburgh Sleep Quality Index (PSQI), developed by Buysse et al. [12], was used. The PSQI is a widely applied instrument for measuring subjective sleep quality over the previous month and has been extensively used in epidemiological and clinical research. Due to its broad use in population-based and clinical studies, it remains one of the standard approaches for assessing sleep quality across different settings [13,14].

The PSQI comprises 19 self-reported items, which are combined into seven components: (1) subjective sleep quality, (2) sleep latency, (3) sleep duration, (4) habitual sleep efficiency, (5) sleep disturbances, (6) use of sleep medication, and (7) daytime dysfunction. Each component is scored from 0 to 3, where higher scores indicate worse sleep in the respective domain. The global PSQI score is calculated as the sum of the seven component scores and ranges from 0 to 21, with higher values reflecting poorer overall sleep quality.

In accordance with the conventional interpretation of the instrument, a global PSQI score >5 was used to define poor sleep quality. In addition to the binary outcome, the global PSQI score and the seven component scores were examined descriptively to characterize the pattern of sleep problems in the study population.

The PSQI was analyzed primarily using the global score as an established measure of subjective sleep quality. Because the factor structure of the PSQI has not, to our knowledge, been specifically validated in Central Asian populations, we did not derive or interpret latent factor-based PSQI subdomains. Component-level analyses were used descriptively according to the standard PSQI scoring structure.

### 4.6. Statistical Analysis

The analysis was conducted in IBM SPSS Statistics version 24.0 using the Complex Samples module to account for the multistage stratified cluster design and sampling weights. The complex sampling design incorporated sampling weights, stratification variables, and clustering at the PSU level; standard errors and 95% confidence intervals were estimated using Taylor series linearization.

Descriptive statistics are presented as unweighted absolute counts (n) and weighted estimates (percentages or means) with 95% confidence intervals. Descriptive analyses included estimation of the weighted prevalence of poor sleep quality, the mean global PSQI score, and the overall distribution of the seven PSQI component scores. Internal consistency of the PSQI component structure was assessed using Cronbach’s alpha calculated across the seven PSQI component scores among participants with complete PSQI data.

For bivariate comparisons, design-adjusted procedures were applied: differences in mean scores were assessed using design-corrected F tests, and distributions of categorical variables were compared using the Rao–Scott χ^2^ test. Statistical significance was defined as *p* < 0.05 using a two-sided criterion.

To identify factors associated with poor sleep quality, a design-adjusted multivariable logistic regression model was fitted with poor sleep quality (global PSQI score > 5) as the binary outcome. All covariates were entered simultaneously to estimate mutually adjusted associations. Results are presented as adjusted odds ratios (aORs) with 95% confidence intervals (CIs) and *p*-values.

Covariate selection was based on a priori theoretical considerations regarding the relationships between socio-demographic, behavioural, and clinical characteristics and sleep quality. Age and sex were treated as baseline factors, socioeconomic variables as structural determinants, and behavioural and clinical variables as health-related characteristics potentially located along intermediate pathways. Given the cross-sectional design, all reported estimates represent adjusted associations rather than causal effects. Given the number of tested associations, interpretation focused primarily on effect sizes and confidence intervals rather than isolated *p*-values.

For descriptive geographic visualization, the weighted prevalence of poor sleep quality was additionally estimated for each administrative unit of Kazakhstan and displayed on a map created using Datawrapper (https://www.datawrapper.de/ (accessed on 15 March 2026)).

Complete responses to all PSQI items required for calculation of the global PSQI score were available for 5872 participants. Analyses of poor sleep quality (PSQI > 5), the global PSQI score, and the PSQI component scores were restricted to respondents with sufficient data to derive the corresponding sleep measures. Multivariable regression was conducted using a complete-case approach and included only participants with complete data on the outcome and all covariates entered into the model, yielding a final regression sample of 5465. Thus, 407 participants with complete PSQI data were excluded from the regression analysis because of missing information on one or more covariates. No imputation was performed.

As a sensitivity analysis, we refitted the multivariable logistic regression model among all participants with complete PSQI data by retaining missing covariate values as separate “missing” categories for categorical predictors. This analysis was performed to assess whether the main findings were robust to the complete-case restriction.

## 5. Conclusions

This nationally representative cross-sectional study showed that poor sleep quality is common among adults in Kazakhstan, affecting 28.1% of the population, and is mainly characterized by sleep latency, subjective sleep quality, and sleep disturbances. Poor sleep quality was independently associated with female sex, older age, diabetes, current smoking, heavy episodic drinking, depressive symptoms, urban residence, and regional differences. These findings provide a national post-transition baseline for sleep health surveillance in Kazakhstan and indicate the need for longitudinal studies to monitor temporal changes, clarify regional patterns, and determine whether future changes in sleep quality are related to social, occupational, environmental, or policy-level factors.

## Figures and Tables

**Table 1:** Socio-demographic, behavioural and clinical characteristics of the study population (*n* = 6712).

Characteristic	Category	*n*	% (95% CI), Weighted
Sex	Male	3196	47.6 (46.4–48.8)
	Female	3516	52.4 (51.2–53.6)
Age group	18–24	911	12.2 (11.4–13.0)
	25–34	1433	20.8 (19.8–21.8)
	35–44	1636	24.9 (23.9–26.0)
	45–54	1226	18.9 (18.0–19.9)
	≥55	1506	23.1 (22.1–24.2)
Ethnicity	Turkic	5159	76.0 (74.9–77.0)
	Slavic	1191	18.5 (17.6–19.5)
	Other	362	5.5 (5.0–6.1)
Marital status	Married/cohabiting	4114	63.9 (62.7–65.1)
	Single	2499	36.1 (34.9–37.3)
	Missing	99	-
Education	Primary	28	0.4 (0.3–0.6)
	Secondary	2760	41.0 (39.8–42.2)
	Higher	3886	58.6 (57.4–59.8)
	Missing	38	-
Occupation	Public sector	1411	21.6 (20.6–22.7)
	Private sector	3514	53.8 (52.6–55.0)
	Students	275	3.6 (3.2–4.1)
	Homemakers	419	5.7 (5.2–6.3)
	Pensioners	640	9.9 (9.2–10.7)
	Unemployed	369	5.3 (4.7–5.8)
	Missing	84	-
Place of residence	Urban	4194	63.0 (61.8–64.2)
	Rural	2447	37.0 (35.8–38.2)
	Missing	71	-
Region of residence	North	1069	16.5 (15.6–17.4)
	Central	479	7.0 (6.4–7.7)
	East	465	7.0 (6.4–7.7)
	South	1990	29.8 (28.7–31.0)
	West	1031	15.2 (14.3–16.1)
	Astana city	502	7.3 (6.7–8.0)
	Almaty city	791	11.5 (10.7–12.3)
	Shymkent city	385	5.7 (5.2–6.3)
BMI	Underweight	210	2.1 (1.8–2.5)
	Normal weight	2483	31.4 (30.3–32.6)
	Overweight	2392	37.3 (36.2–38.5)
	Obesity	1538	29.2 (28.0–30.3)
	Missing	89	-
Blood pressure status	Normotensive	2738	37.4 (36.2–38.5)
	Pre-hypertensive	2251	34.9 (33.7–36.1)
	Hypertensive	1718	27.7 (26.7–28.9)
	Missing	5	-
Diabetes status	No diabetes	5958	87.8 (87.0–88.6)
	Diabetes	754	12.2 (11.4–13.0)
Current smoking	Yes	1552	24.4 (23.4–25.5)
	No	5110	75.6 (74.5–76.6)
	Missing	50	-
HED	No	6069	89.8 (89.1–90.5)
	Yes	643	10.2 (9.5–10.9)
Depressive symptoms	No	5720	88.6 (87.7–89.3)
	Yes	781	11.4 (10.7–12.3)
	Missing	211	-
Physical activity level	Sufficient	4365	66.5 (65.4–67.7)
	Insufficient	2197	33.5 (32.3–34.6)
	Missing	150	-

**Table 2:** Distribution of global PSQI score and poor sleep quality among participants with complete PSQI data.

Indicator	Value
Participants with complete PSQI data, *n* (%)	5872 (87.5)
Global PSQI score, weighted mean	4.43
95% CI for weighted mean	4.36–4.50
Median (IQR), unweighted	4 (3–6)
Good sleep quality (PSQI ≤ 5), *n* (weighted %)	4211 (71.9)
Poor sleep quality (PSQI > 5), *n* (weighted %)	1661 (28.1)

**Table 3:** Structure of PSQI domains.

PSQI Component	Mean Score	95% CI	Any Problem (≥1), %	Moderate/Severe (≥2), %	Share of Summed Mean Score, %
Subjective sleep quality	0.82	0.80–0.83	69.1	11.0	18.4
Sleep latency	0.87	0.85–0.90	63.2	18.8	19.7
Sleep disturbances	0.80	0.79–0.81	73.9	5.9	18.1
Sleep duration	0.66	0.64–0.68	54.6	8.8	14.8
Habitual sleep efficiency	0.59	0.57–0.62	25.5	18.2	13.4
Daytime dysfunction	0.58	0.56–0.60	43.0	12.4	13.1
Use of sleep medication	0.11	0.10–0.12	6.9	2.5	2.4

**Table 4:** Bivariate prevalence of poor sleep quality according to socio-demographic and regional characteristics.

Variable	Category	*n*	Poor Sleep %	95% CI	*p*
Sex	Male	2803	24.7	23.1–26.3	<0.001
	Female	2983	32.1	30.4–33.9	
Age group	18–24	810	21.5	18.6–24.4	<0.001
	25–34	1233	24.8	22.3–27.2	
	35–44	1420	26.7	24.4–29.1	
	45–54	1074	29.5	26.7–32.3	
	55+	1304	35.0	32.3–37.6	
Education	Higher	3355	27.9	26.4–29.5	0.805
	Secondary	2422	28.3	26.5–30.2	
	Primary	28	23.2	7.2–39.2	
Place of residence	Urban	3654	32.2	30.6–33.7	<0.001
	Rural	2131	20.8	19.1–22.6	
Macro-region	North	838	24.1	21.1–27.0	<0.001
	Central	423	39.1	34.4–43.9	
	East	454	37.7	33.2–42.3	
	South	1801	20.9	18.9–22.8	
	West	874	28.2	25.2–31.3	
	Astana city	317	26.6	21.6–31.5	
	Almaty city	757	43.6	40.0–47.2	
	Shymkent city	377	18.6	14.6–22.6	

Analyses were restricted to participants with complete PSQI data and non-missing values for the corresponding variable; therefore, denominators vary across characteristics. Primary education included only 28 participants; estimates for this category should be interpreted cautiously because of sparse data.

**Table 5:** Bivariate prevalence of poor sleep quality according to clinical and behavioural characteristics.

Variable	Category	*n*	Poor Sleep %	95% CI	*p*
BMI	Underweight	176	30.1	23.3–36.9	0.004
	Normal	2177	26.9	25.0–28.8	
	Overweight	2087	26.3	24.4–28.2	
	Obesity	1351	31.3	28.8–33.8	
Blood pressure status	Normotensive	2391	27.6	25.8–29.4	<0.001
	Pre-hypertensive	1965	23.7	21.8–25.6	
	Hypertensive	1481	34.4	31.9–36.9	
Diabetes status	No diabetes	5207	26.4	25.2–27.6	<0.001
	Diabetes	634	40.8	36.9–44.7	
Current smoking	No	4460	27.2	25.8–28.5	0.004
	Yes	1363	31.0	28.5–33.6	
HED	No	5286	27.1	25.8–28.3	<0.001
	Yes	555	37.3	33.2–41.4	
Depressive symptoms	No	5005	23.8	22.6–25.0	<0.001
	Yes	662	59.7	55.9–63.5	
Physical activity	Sufficient	4243	29.1	27.7–30.5	0.004
	Insufficient	1592	25.4	23.2–27.6	

Analyses were restricted to participants with complete PSQI data and non-missing values for the corresponding variable; therefore, denominators vary across characteristics.

**Table 6:** Multivariable logistic regression for poor sleep quality (PSQI > 5) (*n* = 5465).

Variable	Category	aOR (95% CI)	*p*
Sex	Male	Ref	-
	Female	1.37 (1.19–1.57)	<0.001
Age group	18–24	Ref	-
	25–34	1.15 (0.90–1.47)	0.279
	35–44	1.30 (1.02–1.66)	0.037
	45–54	1.47 (1.14–1.90)	0.003
	55+	1.98 (1.53–2.55)	<0.001
Education	Higher	Ref	-
	Secondary	1.09 (0.95–1.24)	0.226
	Primary	1.24 (0.49–3.12)	0.655
Place of residence	Urban	Ref	-
	Rural	0.71 (0.61–0.84)	<0.001
Macro-region	North	Ref	-
	Central	2.00 (1.46–2.74)	<0.001
	East	1.94 (1.48–2.53)	<0.001
	South	1.07 (0.86–1.33)	0.537
	West	1.48 (1.17–1.88)	0.001
	Astana city	1.12 (0.82–1.53)	0.459
	Almaty city	2.18 (1.72–2.76)	<0.001
	Shymkent city	0.82 (0.59–1.14)	0.236
BMI	Normal	Ref	-
	Underweight	1.25 (0.80–1.94)	0.326
	Overweight	1.00 (0.85–1.17)	0.986
	Obesity	1.14 (0.95–1.36)	0.155
Blood pressure status	Normotensive	Ref	-
	Pre-hypertensive	0.89 (0.76–1.04)	0.141
	Hypertensive	1.12 (0.94–1.34)	0.209
Diabetes status	No diabetes	Ref	-
	Diabetes	1.47 (1.22–1.78)	<0.001
Current smoking	No	Ref	-
	Yes	1.28 (1.10–1.50)	0.002
HED	No	Ref	-
	Yes	1.43 (1.16–1.76)	<0.001
Depressive symptoms	No	Ref	-
	Yes	4.26 (3.52–5.15)	<0.001
Physical activity	Sufficient	Ref	-
	Insufficient	0.97 (0.84–1.12)	0.683

The primary education category was small (*n* = 28), resulting in imprecise estimates and wide confidence intervals.

## Data Availability

All available data was presented within manuscript.

## Supplementary Materials

The following supporting information can be downloaded at: https://www.mdpi.com/article/10.3390/clockssleep8020034/s1, Table S1: Comparison of participants included in the multivariable regression model and those excluded because of missing covariate data among respondents with complete PSQI data. Table S2: Sensitivity analysis for multivariable logistic regression of poor sleep quality (PSQI > 5), retaining missing covariate values as separate categories among participants with complete PSQI data.

## Abbreviations

The following abbreviations are used in this manuscript:

aOR	Adjusted odds ratio
AASM	American Academy of Sleep Medicine
BMI	Body mass index
BP	Blood pressure
CI	Confidence interval
DBP	Diastolic blood pressure
EDTA	Ethylenediaminetetraacetic acid
HbA1c	Glycated hemoglobin
HDL-C	High-density lipoprotein cholesterol
HED	Heavy episodic drinking
IQR	Interquartile range
LDL-C	Low-density lipoprotein cholesterol
MET	Metabolic equivalent of task
PHC	Primary health care
PSU	Primary sampling unit
PSQI	Pittsburgh Sleep Quality Index
SBP	Systolic blood pressure
SOPs	Standard operating procedures
STEPS	STEPwise approach to noncommunicable disease risk-factor surveillance
TG	Triglycerides
UTC	Coordinated Universal Time
WHO	World Health Organization

## References

[1] Ramar K., Malhotra R.K., Carden K.A., Martin J.L., Abbasi-Feinberg F., Aurora R.N., Kapur V.K., Olson E.J., Rosen C.L., Rowley J.A. (2021). Sleep is essential to health: An American Academy of Sleep Medicine position statement. J. Clin. Sleep Med..

[2] Lim D.C., Najafi A., Afifi L., Bassetti C.L.A., Buysse D.J., Han F., Högl B., Melaku Y.A., Morin C.M., Pack A.I. (2023). The need to promote sleep health in public health agendas across the globe. Lancet Public Health.

[3] Wang J., Wu J., Liu J., Meng Y., Li J., Zhou P., Xu M., Yan Q., Li Q., Yin X. (2023). Prevalence of sleep disturbances and associated factors among Chinese residents: A web-based empirical survey of 2019. J. Glob. Health.

[4] Santa Helena E.T., Machado N.B., Sakae R.T., de Sousa C.A., Nunes C.R.O., Völzke H., Ewert R., Markus M.R.P. (2024). Sleep quality and associated factors in adults living in the southern Brazil: A population-based study. Sleep Med. X.

[5] Zhang H.S., Li Y., Mo H.Y., Qiu D.X., Zhao J., Luo J.L., Lin W.Q., Wang J.J., Wang P.X. (2017). A community-based cross-sectional study of sleep quality in middle-aged and older adults. Qual. Life Res..

[6] Kyung M., Park S., Park C.G., Hong O. (2024). Association between sleep duration, social jetlag, and the metabolic syndrome by shift works. Int. J. Environ. Res. Public Health.

[7] Rishi M.A., Cheng J.Y., Strang A.R., Sexton-Radek K., Ganguly G., Licis A., Flynn-Evans E.E., Berneking M.W., Bhui R., Creamer J. (2024). Permanent standard time is the optimal choice for health and safety: An American Academy of Sleep Medicine position statement. J. Clin. Sleep Med..

[8] gov.egov.kz In Accordance with the Resolution of the Government of the Republic of Kazakhstan Dated 19 January 2024 No. 20 “on the Procedure for Calculating Time in the Territory of the Republic of Kazakhstan” from 29 February 2024 on the Night of 1 March 2024 (at 00:00), Changes Were Made Providing for a Time Shift Back by 1 Hour. https://www.gov.kz/memleket/entities/kegen-saty/press/news/details/698950?lang=en.

[9] gov.egov.kz Kazakhstan Scientists and Experts Propose to Establish a Single Time Zone in the Country. https://www.gov.kz/memleket/entities/mti/press/news/details/663373?lang=en.

[10] Shinalieva K., Kasenova A., Akhmetzhanova Z., Alzhanova D., Eszhanova L., Bekenova A. (2023). Association of insomnia with anxiety and depression in type 2 diabetic patients: A cross-sectional study. Iran. J. Med. Sci..

[11] Kussainova D.K., Orazalina A.S., Khismetova Z.A., Serikova-Esengeldina D., Khamidullina Z.G., Akhmetova K.M., Tursynbekova A.E., Tukinova A.R., Shalgumbayeva G.M. (2025). Prevalence of anxiety, depression, and insomnia among medical workers in emergency medical services in Eastern Kazakhstan. Int. J. Environ. Res. Public Health.

[12] Buysse D.J., Reynolds C.F., Monk T.H., Berman S.R., Kupfer D.J. (1989). The Pittsburgh Sleep Quality Index: A new instrument for psychiatric practice and research. Psychiatry Res..

[13] Carpi M. (2025). The Pittsburgh Sleep Quality Index: A brief review. Occup. Med..

[14] Mollayeva T., Thurairajah P., Burton K., Mollayeva S., Shapiro C.M., Colantonio A. (2016). The Pittsburgh sleep quality index as a screening tool for sleep dysfunction in clinical and non-clinical samples: A systematic review and meta-analysis. Sleep Med. Rev..

[15] Dunleavy G., Bajpai R., Tonon A.C., Chua A.P., Cheung K.L., Soh C.K., Christopoulos G., de Vries H., Car J. (2019). Examining the factor structure of the Pittsburgh Sleep Quality Index in a multi-ethnic working population in Singapore. Int. J. Environ. Res. Public Health.

[16] Hinz A., Glaesmer H., Brähler E., Löffler M., Engel C., Enzenbach C., Hegerl U., Sander C. (2017). Sleep quality in the general population: Psychometric properties of the Pittsburgh Sleep Quality Index, derived from a German community sample of 9284 people. Sleep Med..

[17] Simonelli G., Marshall N.S., Grillakis A., Miller C.B., Hoyos C.M., Glozier N. (2018). Sleep health epidemiology in low- and middle-income countries: A systematic review and meta-analysis of the prevalence of poor sleep quality and sleep duration. Sleep Health.

[18] Okubo N., Matsuzaka M., Takahashi I., Sawada K., Sato S., Akimoto N., Umeda T., Nakaji S. (2014). Relationship between self-reported sleep quality and metabolic syndrome in general population. BMC Public Health.

[19] Sun X.H., Ma T., Yao S., Chen Z.K., Xu W.D., Jiang X.Y., Wang X.F. (2020). Associations of sleep quality and sleep duration with frailty and pre-frailty in an elderly population Rugao longevity and ageing study. BMC Geriatr..

[20] Doi Y., Minowa M., Uchiyama M., Okawa M. (2001). Subjective sleep quality and sleep problems in the general Japanese adult population. Psychiatry Clin. Neurosci..

[21] Wu W., Jiang Y., Wang N., Zhu M., Liu X., Jiang F., Zhao G., Zhao Q. (2020). Sleep quality of Shanghai residents: Population-based cross-sectional study. Qual. Life Res..

[22] Baglioni C., Battagliese G., Feige B., Spiegelhalder K., Nissen C., Voderholzer U., Lombardo C., Riemann D. (2011). Insomnia as a predictor of depression: A meta-analytic evaluation of longitudinal epidemiological studies. J. Affect. Disord..

[23] Chang K.J., Son S.J., Lee Y., Back J.H., Lee K.S., Lee S.J., Chung Y.K., Lim K.Y., Noh J.S., Kim H.C. (2014). Perceived sleep quality is associated with depression in a Korean elderly population. Arch. Gerontol. Geriatr..

[24] Borzouei S., Ahmadi A., Pirdehghan A. (2024). Sleep quality and glycemic control in adults with type 2 diabetes mellitus. J. Fam. Med. Prim. Care.

[25] Liao Y., Xie L., Chen X., Kelly B.C., Qi C., Pan C., Yang M., Hao W., Liu T., Tang J. (2019). Sleep quality in cigarette smokers and nonsmokers: Findings from the general population in central China. BMC Public Health.

[26] Strüven A., Schlichtiger J., Hoppe J.M., Thiessen I., Brunner S., Stremmel C. (2025). The impact of alcohol on sleep physiology: A prospective observational study on nocturnal resting heart rate using smartwatch technology. Nutrients.

[27] Rahe C., Czira M.E., Teismann H., Berger K. (2015). Associations between poor sleep quality and different measures of obesity. Sleep Med..

[28] Lo K., Woo B., Wong M., Tam W. (2018). Subjective sleep quality, blood pressure, and hypertension: A meta-analysis. J. Clin. Hypertens..

[29] Wang Y.H., Huang C.F., Chen L.J., Ku P.W., Stamatakis E. (2025). Prospective associations between occupational physical activity level and sleep disturbances: A five-year follow-up study. BMC Public Health.

[30] Leota J., Presby D.M., Le F., Czeisler M.É., Mascaro L., Capodilupo E.R., Wiley J.F., Drummond S.P.A., Rajaratnam S.M.W., Facer-Childs E.R. (2025). Dose-response relationship between evening exercise and sleep. Nat. Commun..

[31] Mierniczek M., Mika W., Mierniczek M., Kotte K., Gloc E., Dmowska D., Bilińska A., Zdebski P., Mierniczek A. (2025). Move to sleep: How exercise timing and intensity shape sleep quality—A narrative review. J. Educ. Health Sport.

[32] Xu Y.X., Zhang J.H., Tao F.B., Sun Y. (2023). Association between exposure to light at night (LAN) and sleep problems: A systematic review and meta-analysis of observational studies. Sci. Total Environ..

[33] Carvalho F.G., Hidalgo M.P., Levandovski R. (2014). Differences in circadian patterns between rural and urban populations: An epidemiological study in countryside. Chronobiol. Int..

[34] Fernandes G.L., da Silva Vallim J.R., D’Almeida V., Tufik S., Andersen M.L. (2024). The effects of social jetlag and sleep variability on sleepiness in a population-based study: The mediating role of sleep debt. J. Sleep. Res..

[35] Riley L., Guthold R., Cowan M., Savin S., Bhatti L., Armstrong T., Bonita R. (2016). The World Health Organization STEPwise approach to noncommunicable disease risk-factor surveillance: Methods, challenges, and opportunities. Am. J. Public Health.

