# Supplementary material for: Sleep Quality and Its Sociodemographic, Behavioural, Clinical, and Regional Correlates Among Adults in Kazakhstan: A National Cross-Sectional Survey
Source: Clocks Sleep. 2026 Jun 12;8(2):34. doi: 10.3390/clockssleep8020034 (PMC13298374; doi:10.3390/clockssleep8020034)
Supplement: Supplementary file 1 [file clockssleep-08-00034-s001.zip › clockssleep-4309895-supplementary.pdf]

**Supplementary**

**Table S1.** Comparison of participants included in the multivariable regression model and those excluded because of missing covariate data among respondents with complete PSQI data.

| Characteristic | Category | Included in regression n=5465, n (%) | Excluded from regression n=407, n (%) |
|---|---|---|---|
| Sex | Male | 2645 (48.4) | 169 (41.5) |
| | Female | 2820 (51.6) | 183 (45.0) |
| | Missing | 0 (0.0) | 55 (13.5) |
| Age group | 18-24 | 767 (14.0) | 45 (11.1) |
| | 25-34 | 1136 (20.8) | 107 (26.3) |
| | 35-44 | 1344 (24.6) | 82 (20.1) |
| | 45-54 | 1010 (18.5) | 68 (16.7) |
| | 55+ | 1208 (22.1) | 105 (25.8) |
| Place of residence | Urban | 3411 (62.4) | 264 (64.9) |
| | Rural | 2054 (37.6) | 86 (21.1) |
| | Missing | 0 (0.0) | 57 (14.0) |
| Education | Primary | 27 (0.5) | 1 (0.2) |
| | Secondary | 2277 (41.7) | 157 (38.6) |
| | Higher | 3161 (57.8) | 213 (52.3) |
| | Missing | 0 (0.0) | 36 (8.8) |
| Macro-region | North | 805 (14.7) | 33 (8.1) |
| | Central | 283 (5.2) | 145 (35.6) |
| | East | 442 (8.1) | 15 (3.7) |
| | South | 1701 (31.1) | 110 (27.0) |
| | West | 813 (14.9) | 63 (15.5) |
| | Astana city | 312 (5.7) | 5 (1.2) |
| | Almaty city | 745 (13.6) | 22 (5.4) |
| | Shymkent city | 364 (6.7) | 14 (3.4) |
| Diabetes status | No diabetes | 4874 (89.2) | 360 (88.5) |
| | Diabetes | 591 (10.8) | 47 (11.5) |
| Current smoking | No | 4172 (76.3) | 313 (76.9) |
| | Yes | 1293 (23.7) | 76 (18.7) |
| | Missing | 0 (0.0) | 18 (4.4) |
| HED | No | 4933 (90.3) | 381 (93.6) |
| | Yes | 532 (9.7) | 26 (6.4) |
| Depressive symptoms | No | 4821 (88.2) | 212 (52.1) |
| | Yes | 644 (11.8) | 21 (5.2) |
| | Missing | 0 (0.0) | 174 (42.8) |
| Physical activity | Sufficient | 3967 (72.6) | 298 (73.2) |
| | Insufficient | 1498 (27.4) | 103 (25.3) |
| | Missing | 0 (0.0) | 6 (1.5) |
| Poor sleep quality | Good sleep quality | 3945 (72.2) | 266 (65.4) |
| | Poor sleep quality | 1520 (27.8) | 141 (34.6) |

**Table S2. Sensitivity analysis for multivariable logistic regression of poor sleep quality (PSQI >5), retaining missing covariate values as separate categories among participants with complete PSQI data.**

| Variable | Category | aOR (95% CI) | p-value |
|---|---|---|---|
| Sex | Male | Ref | - |
| | Female | 1.39 (1.21-1.59) | <0.001 |
| Age group | 18-24 | Ref | - |
| | 25-34 | 1.23 (0.97-1.55) | 0.088 |
| | 35-44 | 1.34 (1.06-1.69) | 0.015 |
| | 45-54 | 1.47 (1.15-1.88) | 0.002 |
| | 55+ | 1.95 (1.53-2.49) | <0.001 |
| Education | Higher | Ref | - |
| | Secondary | 1.08 (0.95-1.23) | 0.255 |
| | Primary | 1.01 (0.39-2.62) | 0.978 |
| | Missing | 1.19 (0.56-2.53) | 0.657 |
| Place of residence | Urban | Ref | - |
| | Rural | 0.70 (0.60-0.82) | <0.001 |
| | Missing | 1.30 (0.72-2.35) | 0.381 |
| Macro-region | North | Ref | - |
| | Central | 2.04 (1.52-2.72) | <0.001 |
| | East | 1.94 (1.49-2.52) | <0.001 |
| | South | 1.01 (0.82-1.25) | 0.922 |
| | West | 1.42 (1.13-1.80) | 0.003 |
| | Astana city | 1.07 (0.78-1.46) | 0.694 |
| | Almaty city | 2.07 (1.64-2.61) | <0.001 |
| | Shymkent city | 0.80 (0.58-1.11) | 0.185 |
| BMI | Normal | Ref | - |
| | Underweight | 1.21 (0.84-1.76) | 0.312 |
| | Overweight | 0.98 (0.84-1.14) | 0.820 |
| | Obesity | 1.12 (0.94-1.33) | 0.215 |
| | Missing | 1.89 (1.12-3.19) | 0.016 |
| Blood pressure status | Normotensive | Ref | - |
| | Pre-hypertensive | 0.92 (0.79-1.07) | 0.286 |
| | Hypertensive | 1.22 (1.03-1.46) | 0.022 |
| | Missing | 0.32 (0.03-3.24) | 0.337 |
| Diabetes status | No diabetes | Ref | - |
| | Diabetes | 1.48 (1.22-1.79) | <0.001 |
| Current smoking | No | Ref | - |
| | Yes | 1.30 (1.12-1.52) | 0.001 |
| | Missing | 0.89 (0.26-3.09) | 0.856 |
| Heavy episodic drinking | No | Ref | - |
| | Yes | 1.38 (1.13-1.69) | 0.002 |
| Depressive symptoms | No | Ref | - |
| | Yes | 4.43 (3.70-5.31) | <0.001 |
| | Missing | 1.40 (0.96-2.03) | 0.081 |
| Physical activity | Sufficient | Ref | - |
| | Insufficient | 0.97 (0.84-1.12) | 0.636 |
| | Missing | 0.35 (0.03-3.56) | 0.377 |

aOR, adjusted odds ratio; CI, confidence interval; BMI, body mass index; PSQI, Pittsburgh Sleep Quality Index. The sensitivity analysis included participants with complete PSQI data and retained missing covariate values as separate "Missing" categories for categorical predictors. The model was adjusted for sex, age group, education, place of residence, macro-region, BMI, blood pressure status, diabetes status, current smoking, heavy episodic drinking, depressive symptoms, and physical activity.
